# Serum Vitamin Profile in Oral Lichen Planus Patients in Southwest of Iran

**DOI:** 10.1155/2021/8627435

**Published:** 2021-02-24

**Authors:** Fahimeh Rezazadeh, Sara Haghighat

**Affiliations:** ^1^Oral & Dental Disease Research Center, Department of Oral and Maxillofacial Medicine, School of Dentistry, Shiraz University of Medical Sciences, Shiraz, Iran; ^2^Student Research Committee, School of Dentistry, Shiraz University of Medical Sciences, Shiraz, Iran

## Abstract

**Introduction:**

Oral lichen planus (OLP) is a chronic mucocutaneous disease. It is mainly an immune system-related disorder. Vitamins can modulate immune system functions, and thus, vitamin deficiency might have roles in exacerbating OLP. We aim to determine the serum levels of vitamins A, B12, C, D3, and E in OLP patients.

**Methods and Materials:**

34 OLP patients referred to Shiraz Dental School entered the study. Blood samples were collected and levels of A, B12, C, D3, and E vitamins were measured in serum. 43 healthy people were also included as the control group. Serum levels of vitamins were measured by HPLC (A, B12, D3, and E) and Kiazist analyzing kit (vitamin C).

**Results:**

Most of the patients were female (62.3%), and the mean age of patients was 48.03 ± 11.57. Serum levels of vitamins A, C, and E were lower in OLP patients in comparison with the healthy group; however, the difference was not significant. Vitamins B12 and D3 were higher in the OLP group but the difference was not significant.

**Conclusion:**

Serum levels of vitamins A, B12, C, D3, and E do not have a significant difference in OLP patients and healthy groups. These vitamins may not have a considerable role in OLP pathogenesis in the southwest of Iran.

## 1. Introduction

Oral lichen planus (OLP) is a chronic mucocutaneous disease that is estimated to involve 0.5-2.2% of the whole population [1]. It has been suggested that the prevalence of this disease is greater in Iranian population [2]. Oral manifestations of lichen planus include reticular, papular, erythematous, plaque-like, ulcerative, and bullous-type lesions [3–5]. It usually happens in the 4-5th decades of life and female : male ratio is approximately 3 : 2 [6–8]. OLP is diagnosed by both clinical (bilateral reticular form) and histological characteristics [8].

The exact cause of this disease remains unknown, but it is mainly assumed as an immune system-related disorder [7, 9, 10]. Therefore, any local or systemic factors which influence the immune system could act as a trigger to initiate disease. Immunological disorders, infectious diseases, increased oxidative stress, and malnutrition could all be considered as triggers of disease [4, 7, 11, 12]. It has been suggested that vitamins and micronutrient deficiencies are also effective in initiating or exacerbating the disease [13–17].

Vitamins are categorized due to their roles in the body. C and E vitamins are antioxidants, which reduce reactive oxygen species (ROS) and the resulting oxidative stress in the body [4, 18]. B group vitamins are necessary for cell growth and body metabolism and are also crucial for healthy bone turnover and cognitive functions [10, 19]. Vitamin D is shown to have an immunomodulatory effect and therapeutic effect on autoimmune disorders [20]. It is also crucial for the remodeling of bone and skin [21, 22]. Retinoids including vitamin A are also proved to have immunomodulatory and anti-inflammatory effects and thus can accelerate the healing process of lesions in the skin and mucosa [1, 23].

Some studies have investigated the possible association between vitamin and micronutrient deficiency and oral diseases, but the reported results are controversial. The role of antioxidant vitamins C and E in OLP has been assessed in some studies. In Kaur et al.'s and Nicolae et al.'s studies, significant difference was observed between OLP patients and healthy groups in terms of vitamin concentration while in Nagao et al.'s study, the difference was not significant [13, 18, 24]. In B group vitamins, Chen et al. and Lin et al. reported significant relationships between vitamin B12 deficiency and the presence of OLP in samples of the Thai population [5, 15]. However, a study in the Iranian population showed no significant difference between the two groups [10]. Vitamin D status was also different in Iranian studies. Bahramian et al. and Seif et al. reported a nonsignificant relationship between vitamin D concentration and OLP presence in the Iranian population while Grimm et al. observed a significantly lower vitamin D receptors in German OLP patients compared to healthy people [20, 25, 26].

Due to aforementioned controversial results and lack of evidence about the complete vitamin profile of OLP patients in the southwest of Iran, we aimed to determine the serum levels of vitamins A, B12, C, D3, and E in oral lichen planus patients referred to the Oral and Maxillofacial Medicine Department of Shiraz Dental School in this study. We also aim to compare the results with healthy people and evaluate their correlation with some parameters such as age, sex, and type of OLP.

## 2. Methods

In this cross-sectional study, 34 patients were selected using the convenience sampling method from patients referred to the Oral Medicine Department of Shiraz Dental School. The study was approved by the research ethics committee of Shiraz University of Medical Sciences (Approval ID: IR.SUMS.DENTAL.REC.1398.046). All participants underwent clinical examination by an oral medicine specialist, and histopathological diagnosis was confirmed by an oral pathologist. Patients with a definitive diagnosis of oral lichen planus who did not receive any systemic or local treatments in the last month were enrolled in this study. All patients were interviewed and were asked to fill out written informed consent forms. Patients were excluded from the study if they were using vitamin supplements in the last 6 months or were on special diets such as vegetarians or vegans. Other exclusion criteria were any nutritional or gastrointestinal disorders or any systemic disease which influences vitamin absorption including diabetes. Lichenoid reactions and lesions with the presence of dysplasia.

The type of lesion (erosive or nonerosive) also was recorded. A 5 mL peripheral venous blood sample was obtained from each patient by an expert nurse. Blood samples of 43 healthy patients (as the control group) were also obtained after filling informed consent forms. The samples were frozen at -70°C until all samples were collected.

All blood samples were sent to a private laboratory for measuring the concentration of vitamins A, B12, C, D3, and E. Specific analyzing kit (Kiazist vitamin C kit, Iran) was used for measuring the concentration of vitamin C in blood samples. The analysis for other vitamins (A, B12, D3, and E) was carried out by High-Performance Liquid Chromatography (HPLC) (Infinity 1260, Agilent, USA), using a RP-C18 column with 10°cm length, 4.6°mm internal diameter, and 5°*μ*m particles. The mobile phase consisted of methanol and water by 80 : 20°*v*/*v*. The flow rate of the mobile phase was 1°mL/min. 20°*μ*L of each sample was injected into the column using a Hamilton microsyringe. The concentration of vitamins was measured using UV detection at various wavelengths: 265°nm for vitamin D, 360°nm for vitamin B12, and 280°nm for vitamins A and E. All HPLC measurements were performed using 1260-online software.

Data were analyzed by *t*-test, Pearson chi-square, and Mann-Whitney tests in SPSS software version 22.0. A *P* value of 0.05 was used as the level of significance.

## 3. Results

77 people were enrolled in this cross-sectional study from which 34 were OLP patients and 43 were healthy people as the control group. The two groups were age- and sex-matched. 62.3% of all participants in this study were female (Table 1). In the OLP group, the mean age of patients was 48.03 ± 11.57 and 67.6% of them were female. 53.6% of patients had nonerosive, and 46.4% had erosive OLP. The concentration of vitamins in different groups is summarized in Table 2.

Pearson chi-square test was used to assess the relationship between sex and the presence of disease, and there were no statistically significant differences between the two groups (*P* = 0.393). Similarly, there was not any significant relationship between age and presence of disease according to the *t*-test (*P* = 0.804).

**Figure 1:**
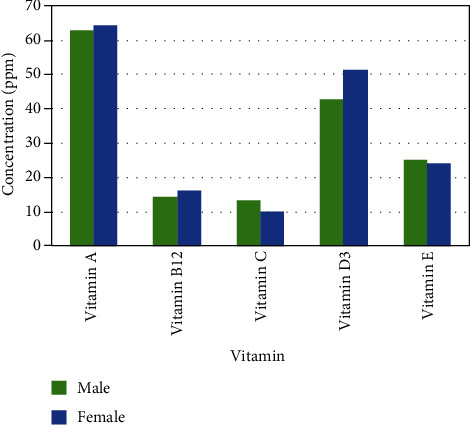
Vitamin concentrations in OLP patients and healthy controls.

Concentrations of vitamins in case and control groups are summarized in Table 2 and Figure 1. Vitamin A, C, and E levels were lower in the OLP group, but vitamin B12 and D3 levels were higher in the patients' group, and neither of differences was significant. Due to the nonnormal distribution of data, the Mann-Whitney test was used to define the significance of this relation.

**Figure 2:**
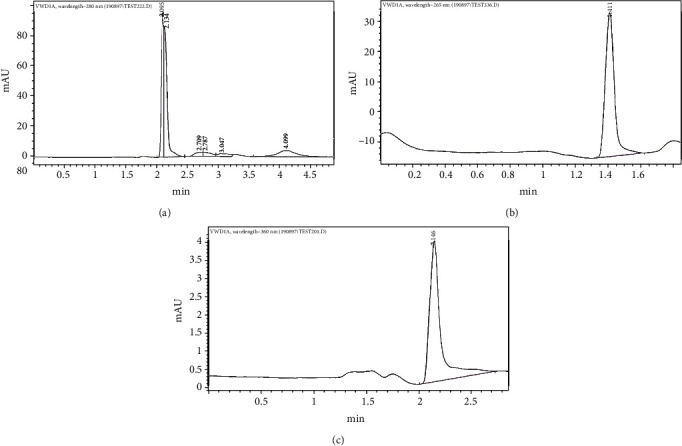
Chromatograms of one of serum samples: (a) vitamins A and E (280 nm); (b) vitamin D (265 nm); (c) vitamin B12 (360 nm).

Serum levels of vitamins were not significantly different with respect to gender (Table 3). Correlation between age and serum levels of vitamins was assessed by Spearman's rho test. A coefficient is also reported for the assessment of the correlation of each vitamin concentration and age (Table 4). Also, relation of severity of disease (different subtypes of OLP including erosive and nonerosive) with vitamin profile was assessed and the difference was not significant. None of the vitamins had a significant age-related correlation. *P* values are recorded in the given tables. Chromatograms of one of the tested samples are given in Figure 2.

## 4. Discussion

In this study, we evaluated serum levels of 5 vitamins in OLP patients and compared them with the healthy group. Most of the patients in this study were female (67.6%) which is consistent with many previous studies [2, 6, 14, 27]. This is probably related to the autoimmune base of disease that is more evident in women than men [28–30]. It could also be explained by the higher referral rate of women to doctors. Some other studies mentioned that the prevalence of OLP in men and women is the same [31, 32]. This controversy might be due to differences in populations or sampling methods.

The mean age of OLP patients in our study was 48.031 ± 11.57 years. This is in accordance with previous studies that reported that the highest incidence rate of OLP is in the 4-5^th^ decade of life [2, 4, 7, 8]. In a recent systematic review and meta-analysis about OLP global status, it is mentioned that the prevalence of OLP is higher in people aged 40 and older [33]. None of the vitamins' concentration was significantly different in male and female groups. We also did not find any significant correlation between age and concentration of any of the vitamins. To the best of our knowledge, none of the earlier studies reported the age correlation and therefore, we had no data to compare to ours.

Due to the specific roles of vitamins, we discuss the results of each vitamin status in healthy and OLP patients in detail separately. Vitamin A was evaluated in this study due to its immunomodulatory effect [23, 24]. OLP etiology is suggested to be highly relevant to immune system disorders [2, 27, 28], and therefore, vitamin A deficiency could be also considered a cause of exacerbating OLP manifestations. In the present study, we recorded a lower level of vitamin A in the OLP group but the difference between the two groups was not significant. This is in accordance with Nagao et al.'s study that investigated the concentration of different types of retinoids including *β*-carotene in OLP patients and healthy controls. They reported that the only micronutrient which was significantly different among cases and controls was retinol and other carotenoids were not significantly different [24]. Some other studies evaluated vitamin A in other immune system-related disorders. Szodoray et al. compared serum levels of vitamin A in Sjogren syndrome patients with healthy controls, and there was not a significant difference between the two groups [34]. Furthermore, some interventional studies evaluated the effects of carotenoids in the treatment of OLP and other immune system-related diseases. Sahebjamee and Bakhshi compared vitamin A and triamcinolone in treating OLP and reported unfavorable results with retinoic acid topical use [23]. Also, in a review article about the management of oral and cutaneous lichen planus, it was mentioned that one of the retinoids (acitretin) is suggested as a 3^rd^-choice treatment for OLP [35]. It can be indicated that while retinoids and vitamin A might have a crucial role in exacerbating OLP in patients, there are still controversial results about the difference between OLP patients and the healthy population. Also, vitamin therapy might not be very efficient in the treatment of OLP.

The role of oxidative stress in OLP pathogenesis has been confirmed in different studies [4, 18, 36]. Vitamins C and E could reduce ROS and the resulting damage to the skin and mucosa [32]. They are both considered effective factors in decreasing oxidative stress and thus might be protective against OLP [4, 13]. According to our results, serum levels of these two vitamins were lower in patients but the difference between the two groups was not significant (*P* value = 0.203 and 0.137 for vitamins C and E, respectively). There have been some reports about these two vitamins and other antioxidant status in LP patients. Barikbin et al. and Nicolae et al. investigated levels of vitamin C in serum and urine, respectively, and both reported a significant difference between healthy and lichen planus groups [18, 32]. Kaur et al. and Abdolsamadi et al. evaluated salivary levels of vitamin C and reported a significantly lower concentration in patients [4, 13]. Vitamin E levels were also significantly lower in Abdolsamadi et al.'s study [4]. The difference in significance level in OLP and healthy groups is mostly due to varieties in the sample population and sample size.

In our study, there was not a significant difference between the two groups in terms of vitamin B12 levels. This is in accordance with some previous studies. There have been also reports which showed that vitamin B12 and homocysteine deficiency is more prevalent in OLP patients than healthy controls [5, 37]. Mirzaie et al. evaluated folic acid in Iranian OLP patients and reported that there is not a significant difference between healthy and patient groups [10]. Rahmatpour et al. also reported that vitamin B12 levels in another group of Iranian OLP patients are within the normal range [38]. Thongprasom et al. evaluated homocysteine levels in OLP patients in a Thai group of patients and reported that there was not a significant difference between patients and healthy controls in their study [39]. In contrast, there was another study done by Chen et al. which evaluated serum levels of vitamin B group in a relatively large Chinese population and reported a significantly lower measurement in OLP patients [5]. These studies suggest that vitamin B12 deficiency might not be strongly associated with OLP pathogenesis. Also, there might be a correlation between race and vitamin B12 status. Further studies with larger sample sizes and heterogenic races are needed to confirm this conclusion.

Vitamin D is suggested to have anticancerous and apoptosis-inducing effects. It is also reported that vitamin D levels are severely deficient in oral squamous cell carcinoma and precancerous lesions [26]. In our study, there was not a significant difference in vitamin D levels between cases and controls. This is similar to some previous Iranian studies. Bahramian et al. and Seif et al. evaluated serum levels of vitamin D3 in OLP patients and healthy controls, and the difference was not statistically significant [20, 25]. A recent study in Tehran, Iran, evaluated serum and salivary levels of vitamin D in OLP patients and reported that serum levels of this vitamin do not have a significant difference in comparison to controls. In contrast, salivary levels of vitamin D were significantly lower in OLP patients [40]. This might be due to the upregulation of vitamin D-binding protein (DBP) in inflammatory and immune system-related disorders that causes an increased record of serum levels of vitamin D but does not have an impact on salivary levels [40]. Despite the aforementioned studies in Iran, a study in India reported that the serum levels of vitamin D were significantly lower in OLP patients [41]. This indicates the possible role of race and nutritional habits as effective factors in the pathogenesis of OLP.

In our study, we investigated serum levels of 5 vitamins in OLP patients and healthy controls. To the best of our knowledge, this was the first study that provided a full vitamin level profile in OLP patients in the south of Iran. Because of the relatively high prevalence of OLP in the southwest of Iran [2, 7], findings of this study could help predict possible causes of OLP. We used HPLC for measuring vitamin levels in this study. HPLC is an accurate method for measuring levels of different components in body fluids including serum, blood, and saliva [42–44]. In analyzing our data, we used the correlation coefficient to define the relation between age and serum levels of vitamins and this could help have a better assessment of the effect of age on vitamin status and OLP prevalence.

One of our limitations was our rather small sample size; therefore, the data in our study might be remarkably different from actual values in the population. With a larger sample size, this correlation may be better defined. Specimens were frozen in our study before measuring the concentration of vitamins due to proposed methods in similar previous studies. However, authors guess that this condition might have had an adverse effect on the results as some vitamins are sensitive to temperature changes. Baseline differences in the daily diet of participants could act as a confounder in our results.

For future studies, studies with larger sample sizes and investigations on the fresh specimen are recommended in the Iranian population. Recording details about the baseline diet would be crucial for confirmation of the results. Interventional studies are also recommended to assess the use of vitamin supplements for improving disease signs and symptoms. Moreover, systematic reviews might help compare data in different countries to investigate the possible impact of different nutritional habits and races on the findings.

## 5. Conclusion

Based on our results, although serum levels of vitamins A, C, and E were lower in OLP patients in comparison with the healthy group, the difference was not significant. Vitamins B12 and D were not significantly different in two groups either. Our results indicate that vitamins may not have a crucial role in the development of OLP.

## Figures and Tables

**Table 1:** Description of participants in the study with respect to gender and age.

	Gender		Age
	Female	Male	Mean ± SD
Healthy (control)	25 (58.1%)	18 (41.9%)	48.744 ± 12.70
OLP patients (case)	23 (67.6%)	11 (32.4%)	48.031 ± 11.57

**Table 2:** Serum levels of vitamins (ppm) in case and control groups.

	Healthy	OLP patients			*P* value	
		Erosive	Nonerosive	Total	(healthy vs. OLP patients)	(erosive vs. nonerosive)
Vitamin A	72.31 ± 8.57	55.51 ± 38.84	63.70 ± 43.86	55.13 ± 7.52	0.508	0.419
Vitamin B12	15.07 ± 1.25	18.42 ± 7.33	14.74 ± 11.22	16.00 ± 1.70	0.770	0.168
Vitamin C	12.39 ± 2.02	9.61 ± 3.65	9.50 ± 3.85	9.87 ± 0.77	0.203	0.850
Vitamin D3	46.99 ± 4.27	63.13 ± 29.76	41.49 ± 35.43	50.13 ± 6.47	0.802	0.930
Vitamin E	28.38 ± 2.75	19.44 ± 15.89	23.04 ± 14.31	20.70 ± 3.05	0.137	0.824

*P* values are calculated for mean of total OLP patients.

**Table 3:** Serum levels of vitamins in male and female groups.

	Mean ± S.E.		Median		*P* value
	Male	Female	Male	Female	
Vitamin A	63.04 ± 9.55	64.31 ± 7.35	68.68	53.92	0.966
Vitamin B12	14.41 ± 1.33	16.10 ± 1.42	13.78	15.05	0.838
Vitamin C	13.21 ± 2.99	10.10 ± 0.63	8.64	8.71	0.645
Vitamin D3	42.62 ± 4.92	51.56 ± 5.17	46.58	45.33	0.682
Vitamin E	25.30 ± 3.53	24.25 ± 2.64	24.17	21.63	0.383

*P* values are calculated for mean ranks.

**Table 4:** Correlation between age and vitamin concentration.

	Correlation coefficient	*P* value
Vitamin A	-0.073	0.532
Vitamin B12	0.111	0.343
Vitamin C	0.041	0.774
Vitamin D3	-0.100	0.391
Vitamin E	-0.115	0.325

## Data Availability

The demographic data used to support the findings of this study were supplied by the Dentistry School of Shiraz University of Medical Sciences under license and so cannot be made freely available. Requests for access to these data should be made to Sara Haghighat (haghighatsd@gmail.com).

## References

[1] Dalirsani Z., Taghavi Zenouz A., Mehdipour M., Alavi F., Javadzadeh Y. (2010). Comparison of the effect of combination of triamcinolone acetonide and vitamin A mouthwash with triamcinolone mouthwash alone on oral lichen planus. *Journal of Dental Research Dental Clinics Dental Prospects*.

[2] Pakfetrat A., Javadzadeh-Bolouri A., Basir-Shabestari S., Falaki F. (2009). Oral lichen planus: a retrospective study of 420 Iranian patients. *Medicina Oral, Patología Oral y Cirugía Bucal*.

[3] Wu Y.-C., Wang Y.-P., Chang J. Y.-F., Cheng S.-J., Chen H.-M., Sun A. (2014). Oral manifestations and blood profile in patients with iron deficiency anemia. *Journal of the Formosan Medical Association*.

[4] Abdolsamadi H., Rafieian N., Goodarzi M. T. (2014). Levels of salivary antioxidant vitamins and lipid peroxidation in patients with oral lichen planus and healthy individuals. *Chonnam Medical Journal*.

[5] Chen H.-M., Wang Y.-P., Chang J. Y.-F., Wu Y.-C., Cheng S.-J., Sun A. (2015). Significant association of deficiencies of hemoglobin, iron, folic acid, and vitamin B_12_ and high homocysteine level with oral lichen planus. *Journal of the Formosan Medical Association*.

[6] Chiang C. P., Yu-Fong Chang J., Wang Y. P., Wu Y. H., Lu S. Y., Sun A. (2018). Oral lichen planus - differential diagnoses, serum autoantibodies, hematinic deficiencies, and management. *Journal of the Formosan Medical Association*.

[7] Nosratzehi T. (2018). Oral lichen planus: an overview of potential risk factors, biomarkers and treatments. *Asian Pacific Journal of Cancer Prevention*.

[8] Agha-Hosseini F., Mirzaii-Dizgah I., Farmanbar N., Abdollahi M. (2012). Oxidative stress status and DNA damage in saliva of human subjects with oral lichen planus and oral squamous cell carcinoma. *Journal of Oral Pathology & Medicine*.

[9] Buajeeb W., Kraivaphan P., Amornchat C., Suthamajariya K. (2008). Reduction of micronuclei in oral lichen planus supplemented with beta-carotene. *Journal of Oral Science*.

[10] Mirzaie A. R., Shahzadeh M. H., Barzegari M. (2018). Comparison of serum folic acid level in oral lichen planus patients and healthy subjects. *Journal Research Dentomaxillofac Sciences*.

[11] Moshaverinia M., Rezazadeh F., Dalvand F., Moshaverinia S., Samani S. S. (2014). The relationship between oral lichen planus and blood group antigens. *World Journal of Medical Sciences*.

[12] Rezazadeh F., Shahbazi F., Andisheh-Tadbir A. (2017). Evaluation of salivary level of IL-10 in patients with oral lichen planus, a preliminary investigation. *Comparative Clinical Pathology*.

[13] Kaur J., Politis C., Jacobs R. (2016). Salivary 8-hydroxy-2-deoxyguanosine, malondialdehyde, vitamin C, and vitamin E in oral pre-cancer and cancer: diagnostic value and free radical mechanism of action. *Clinical Oral Investigations*.

[14] Chang J. Y., Chen I. C., Wang Y. P., Wu Y. H., Chen H. M., Sun A. (2016). Anemia and hematinic deficiencies in gastric parietal cell antibody-positive and antibody-negative erosive oral lichen planus patients with thyroid antibody positivity. *Journal of the Formosan Medical Association*.

[15] Lin H. P., Wang Y. P., Chia J. S., Chiang C. P., Sun A. (2011). Modulation of serum gastric parietal cell antibody level by levamisole and vitamin B12 in oral lichen planus. *Oral Diseases*.

[16] Rezazadeh F., Salehi S., Rezaee M. (2019). Salivary level of trace element in oral lichen planus, a premalignant condition. *Asian Pacific Journal of Cancer Prevention*.

[17] Rezazadeh F., Sokhakian M. (2018). Plasma level of trace elements in patients with oral lichen planus. *Iranian Journal of Dermatology*.

[18] Nicolae I., Mitran C. I., Mitran M. I., Ene C. D., Tampa M., Georgescu S. R. (2017). Ascorbic acid deficiency in patients with lichen planus. *Journal of Immunoassay & Immunochemistry*.

[19] Morris M. S. (2012). The role of B vitamins in preventing and treating cognitive impairment and decline. *Advances in Nutrition*.

[20] Bahramian A., Bahramian M., Mehdipour M. (2018). Comparing vitamin D serum levels in patients with oral lichen planus and healthy subjects. *J Dent (Shiraz).*.

[21] Kechichian E., Ezzedine K. (2018). Vitamin D and the skin: an update for dermatologists. *American Journal of Clinical Dermatology*.

[22] Jagelaviciene E., Vaitkeviciene I., Silingaite D., Sinkunaite E., Daugelaite G. (2018). The relationship between vitamin D and periodontal pathology. *Medicina*.

[23] Sahebjamee M., Bakhshi M. A. M. (2004). Efficacy of topical retinoic acid compared with topical triamcinolone acetonide in the treatment of oral lichen planus. *Acta Medica Iranica*.

[24] Nagao T., Warnakulasuriya S., Ikeda N. (2001). Serum antioxidant micronutrient levels in oral lichen planus. *Journal of Oral Pathology & Medicine*.

[25] Seif S., Jafari-Ashkavandi Z., Mardani M., Hamidizadeh N. (2018). Evaluation of serum vitamin D level in oral lichen planus patients. *Journal of Mashhad Dental School*.

[26] Grimm M., Cetindis M., Biegner T. (2015). Serum vitamin D levels of patients with oral squamous cell carcinoma (OSCC) and expression of vitamin D receptor in oral precancerous lesions and OSCC. *Medicina Oral, Patología Oral y Cirugía Bucal*.

[27] Li X. Z., Zhang S. N., Yang X. Y. (2019). Serum-based metabolomics characterization of patients with reticular oral lichen planus. *Archives of Oral Biology*.

[28] Carvalho C. H., Santos B. R., Vieira Cde C., Lima E., Santos P. P., Freitas R. A. (2011). Estudo epidemiológico das doenças dermatológicas imunologicamente mediadas na cavidade oral. *Anais Brasileiros de Dermatologia*.

[29] Lu R., Zhou G., Du G., Xu X., Yang J., Hu J. (2011). Expression of T-bet and GATA-3 in peripheral blood mononuclear cells of patients with oral lichen planus. *Archives of Oral Biology*.

[30] Chen Y. T., Wang Y. H., Yu H. C., Yu C. H., Chang Y. C. (2018). Time trend in the prevalence of oral lichen planus based on Taiwanese National Health Insurance Research Database 1996-2013. *J Dent Sci.*.

[31] Farhi D., Dupin N. (2010). Pathophysiology, etiologic factors, and clinical management of oral lichen planus, part I: facts and controversies. *Clinics in Dermatology*.

[32] Barikbin B., Yousefi M., Rahimi H., Hedayati M., Razavi S. M., Lotfi S. (2011). Antioxidant status in patients with lichen planus. *Clinical and Experimental Dermatology*.

[33] Li C., Tang X., Zheng X. (2020). Global prevalence and incidence estimates of oral lichen planus: a systematic review and meta-analysis. *JAMA Dermatology*.

[34] Szodoray P., Horvath I. F., Papp G. (2010). The immunoregulatory role of vitamins A, D and E in patients with primary Sjogren's syndrome. *Rheumatology (Oxford, England)*.

[35] Manousaridis I., Manousaridis K., Peitsch W. K., Schneider S. W. (2013). Individualizing treatment and choice of medication in lichen planus: a step by step approach. *Journal der Deutschen Dermatologischen Gesellschaft*.

[36] Chakraborthy A., Ramani P., Sherlin H. J., Premkumar P., Natesan A. (2014). Antioxidant and pro-oxidant activity of vitamin C in oral environment. *Indian Journal of Dental Research*.

[37] Challacombe S. J. (1986). Haematological abnormalities in oral lichen planus, candidiasis, leukoplakia and non-specific stomatitis. *International Journal of Oral and Maxillofacial Surgery*.

[38] Rahmatpour Rokni G., Heydari F., Golpour M., Yazdani J., Heidari Gorji A. M. (2017). Evaluation of serum homocysteine levels in patients with cutaneous-oral lichen planus and psoriasis patients. *Galen Medical Journal*.

[39] Thongprasom K., Youngnak P., Aneksuk V. (2001). Folate and vitamin B12 levels in patients with oral lichen planus, stomatitis or glossitis. *The Southeast Asian Journal of Tropical Medicine and Public Health*.

[40] Gholizadeh N., Pirzadeh F., Mirzaii-Dizgah I., Sheykhbahaei N. (2020). Relationship between salivary vitamin D deficiency and oral lichen planus. *Photodermatology, Photoimmunology & Photomedicine*.

[41] Akanksha G., Ravi P. S. M., Nagaraju K., Sangeeta M., Sumit G., Swati G. (2017). Serum vitamin D level in oral lichen planus patients of North India- a case-control study. *Journal Dermatology Research Therapy*.

[42] Ladeira C., Padua M., Veiga L., Viegas S., Carolino E., Gomes M. C. (2016). Influence of serum levels of vitamins A, D, and E as well as vitamin D receptor polymorphisms on micronucleus frequencies and other biomarkers of genotoxicity in workers exposed to formaldehyde. *Journal of Nutrigenetics and Nutrigenomics*.

[43] Sobotka R., Capoun O., Kalousova M., Hanus T., Zima T., Kostirova M. (2017). Prognostic importance of vitamins A, E and retinol-binding protein 4 in renal cell carcinoma patients. *Anticancer Research*.

[44] Huang G. L., Yang L., Su M. (2014). Vitamin D3 and beta-carotene deficiency is associated with risk of esophageal squamous cell carcinoma - results of a case-control study in China. *Asian Pacific Journal of Cancer Prevention*.

